# Supplementary material for: Frictiotaxis underlies focal adhesion-independent durotaxis
Source: Nat Commun. 2025 Apr 23;16:3811. doi: 10.1038/s41467-025-58912-1 (PMC12019219; doi:10.1038/s41467-025-58912-1)
Supplement: Supplementary file 1 — Supplementary Information [file 41467_2025_58912_MOESM1_ESM.pdf]

Supplementary Figures

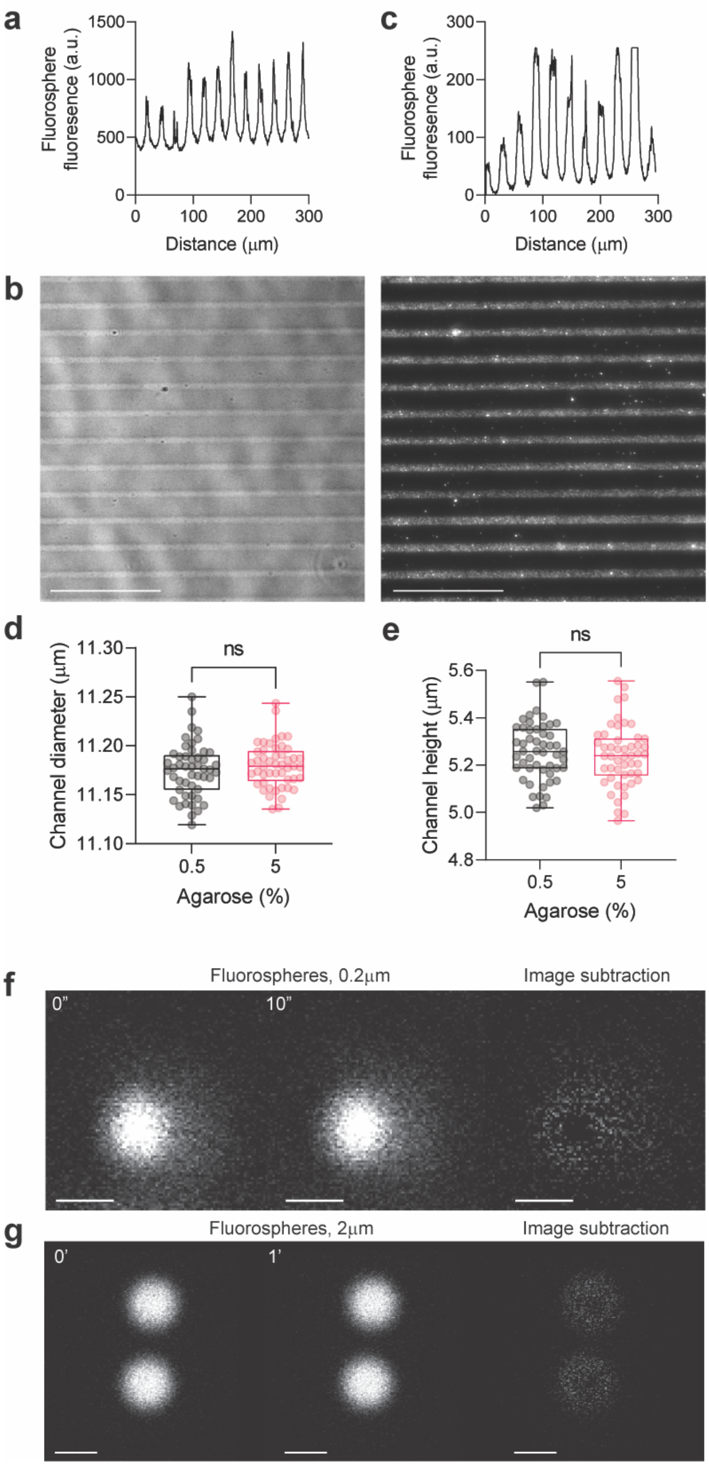

Supplementary Fig 1. The agarose microchannel assay. a-c, 5 μm agarose channels filled with 0.2 μm fluorospheres (**b**) and quantification along the orthogonal axis of fluorosphere-filled 10 μm (**c**) and 5 μm channels (**a**), as in Fig. 1c and Extended Data Fig. 1b, respectively. Scale bar, 100 μm. **d-e**, Channel dimensions quantified. Translucent dots represent individual data points. *N* = 50 channels; unpaired two-tailed t test; ns, $P>0.05$ (exact: $P=0.3482$ in **d**; $P=0.4649$ in **e**). **f-g**, 0.2 μm (**f**) or 2 μm fluorospheres (**g**) in 10 μm agarose microchannels at different time points. The right panel is an image subtraction between the images. Scale bar, 0.5 μm (**f**); 2 μm (**g**).

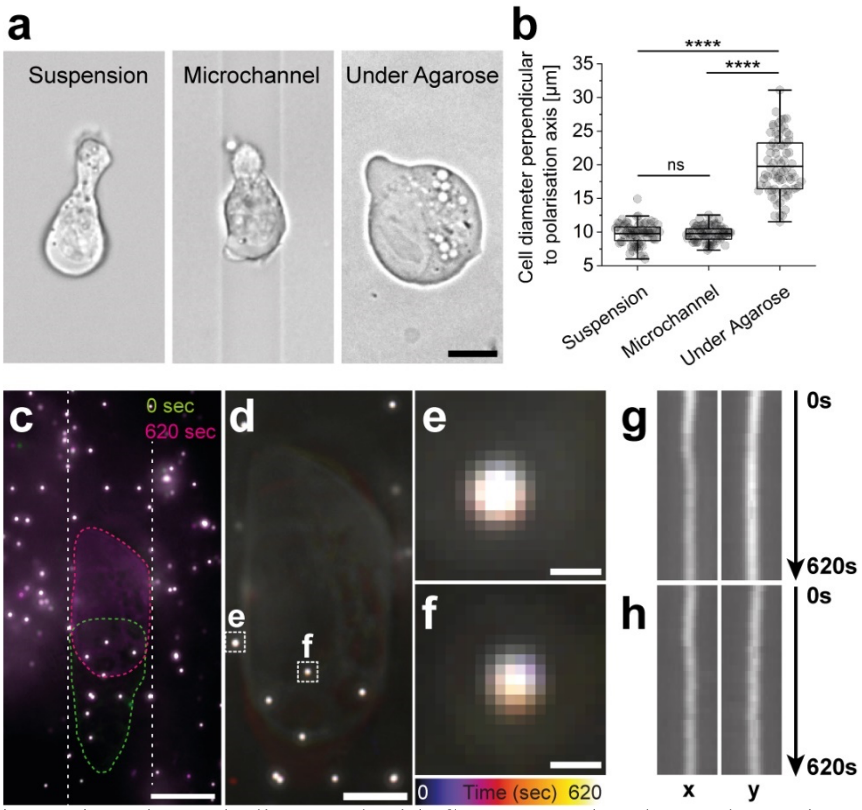

Supplementary Fig 2. Agarose microchannels provide confinement but do not exert compression on Walker cells. a, Walker cells in suspension (left panel), in microchannels (middle panel), and under agarose (right panel). **b**, Quantification of the cell diameter of polarised Walker cells under the depicted conditions. $N =$ 75 cells each; unpaired two-tailed t test; ns, P>0.05 (exact: P=0.97817); ****, P≤0.0001. **c**, Walker cell in a microchannel, dispersed with fluorescent beads, as shown in Supplement Video 1. The focal plane captures interface of the agarose channel and the cell. Individual frames from t=0 sec and t=620 sec were overlayed. White dashed lines mark the channel boundaries, green and pink dashed lines mark the cell at the respective time points. Scale bar, 10 µm. **d**, Color-coded timelapse record of the cell depicted in (**c**). Scale bar, 5 µm. **e**, **f**, Close-ups of individual beads from (**d**), located at the side interface (**e**) and the top interface (**f**) between the cell and the channel walls. Scale bar, 0.5 µm. **g**, **h**, Kymographs from x and y sections of the beads depicted in (**e**) and (**f**).

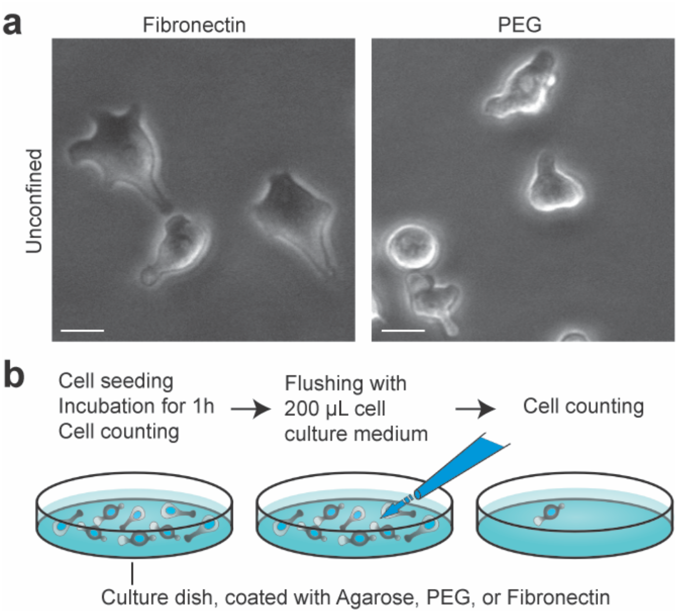

Supplementary Fig 3. Walker cells lack strong and specific adhesions. a, Pictures of cells in adhesive (fibronectin, left) or non-adhesive (PEG, right) conditions. Scale bar, 10 μm. **b**, Illustration depicting the principle and quantification of the flushing experiment.

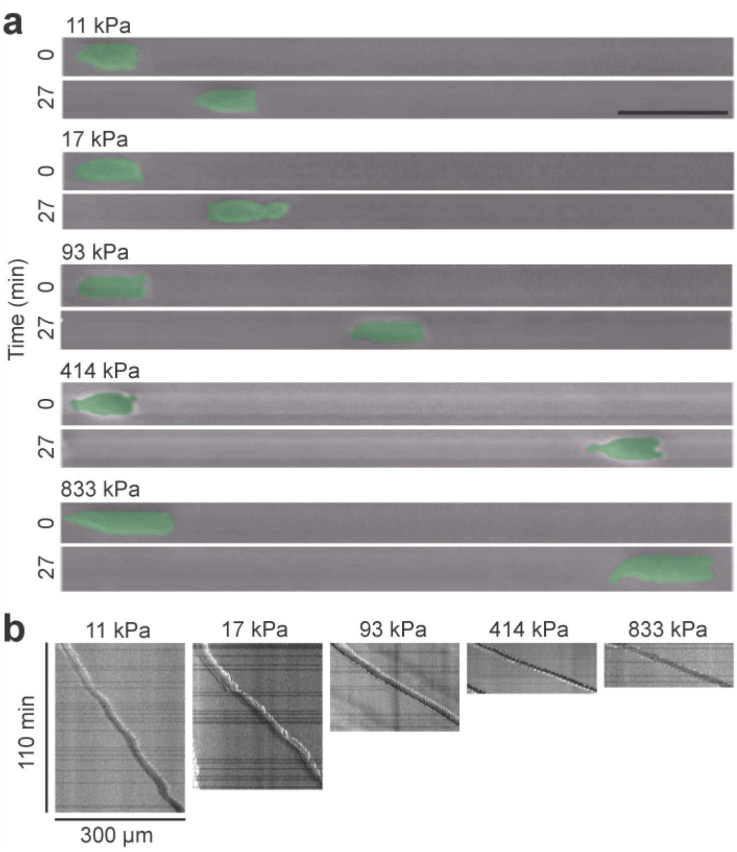

Supplementary Fig 4. Stiffness-dependent migration speed in microchannels. a, Pictures of cells with pseudocoloured membrane at early (t=0 min) and later (t=27 min) time points in microchannels of different stiffness regimes. Scale bar, 50 µm. **b**, Kymographs corresponding to the cells depicted in (**a**). Scale bars equal 300 µm (horizontal) and 110 min (vertical).

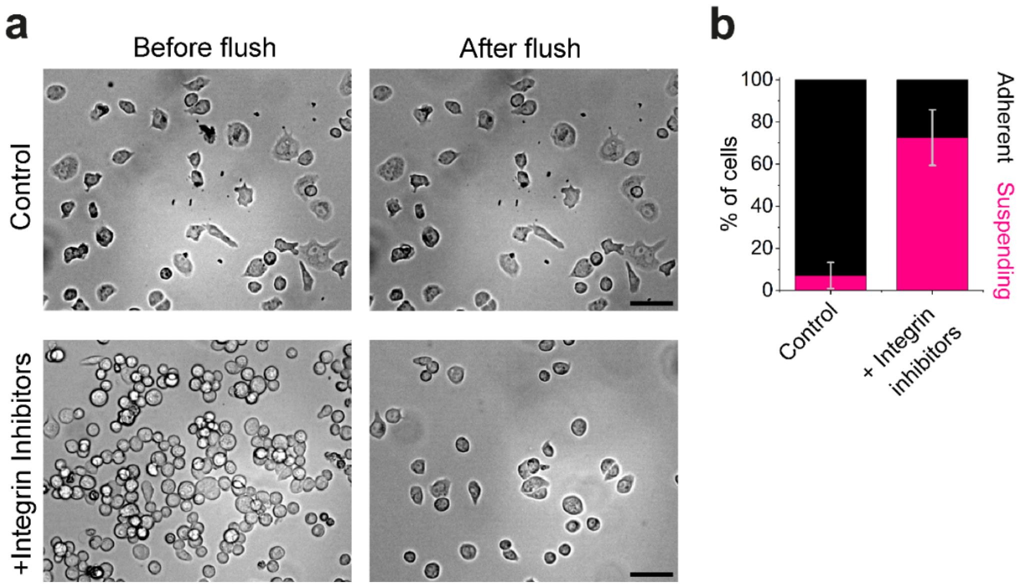

Supplementary Fig 5. A combination of integrin-inhibitors prevents cell adhesion of Walker cells. a, Adhesive subpopulation of Walker cells on FCS-coated coverslips in the absence (top panels) or presence of integrin inhibitors (10 µg mL^{-1} β1-integrin blocking antibody + 10 µM Cilengitide) (bottom panels). Left images shows cells before flushing the substrate, right panels after flushing the substrate with culture media. Scale bar, 50 µm. **b**, Quantification of adherent and flushed fraction of Walker cells after flushing the respective substrates with culture media. Bars represent mean ± s.d.; N = 3 experimental repeats.

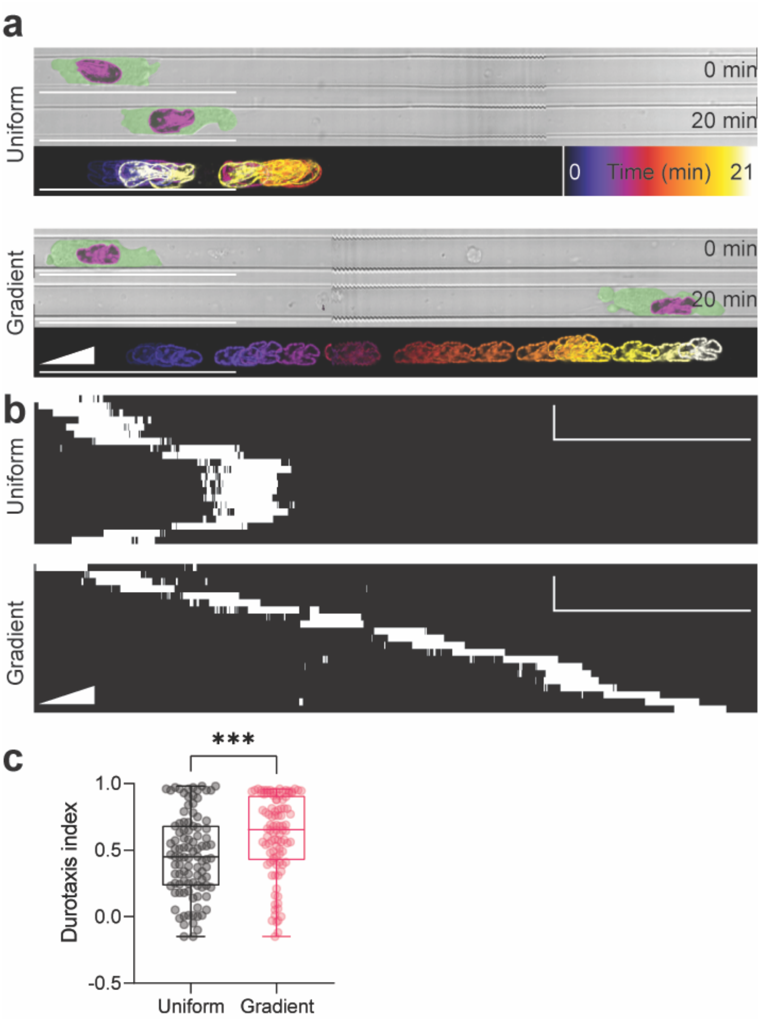

Supplementary Fig 6. Durotaxis of HL60 cells. a-b, Example cells at early and later time points (upper and middle panels, **a**), temporal colour-coded projections (bottom panels, **a**) and kymographs (**b**) in uniform and graded stiffness substrate. Cells were tracked by labelling the nucleus with Hoechst 33342 and the cell membrane was pseudocoloured. Images are stitched as described in the Methods. Scale bar, 50 µm. Vertical scale bar (**b**), 5 min. **c,** Durotaxis quantification. n = 100 cells; two-tailed Mann-Whitney test; ***$P \leq 0.001$ (exact: P=0.0006).

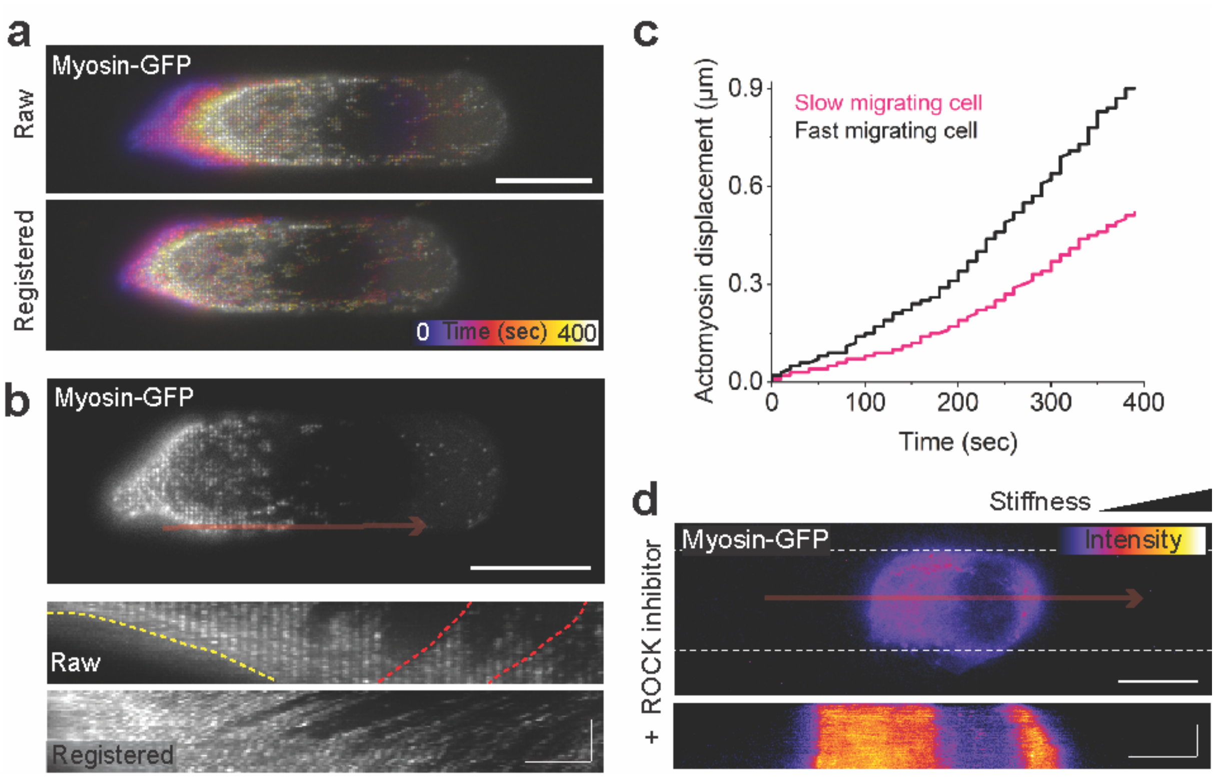

Supplementary Fig 7. Visualisation of retrograde actomyosin flow. a, Temporal colour-coded projection of a Myosin-GFP-expressing Walker cell before (raw top panel) and after image registration (registered bottom panel). Scale bar equals 10 µm. **b**, Still image and kymographs along the area of the red arrow, as shown in Figure 4c, before (Raw) and after image registration (Registered). The yellow dashed line highlights the cell displacement at the rear, while the red dashed lines highlight the retrograde flow of myosin filaments against the direction of migration. Scale bars, 10 µm in the upper panel; 5 µm (horizontal) and 200 s (vertical) in kymographs of lower panels. **c**, Examples of actomyosin displacement over time of a slow (1.47 µm/min) versus faster (2.16 µm/min) migrating cell in a microchannel with stiffness gradient. **d**, Colour-coded still image (upper panel) and Kymograph (lower panel, corresponding to the red arrow in the upper panel) of a Walker cell expressing Myosin-GFP, in a microchannel with stiffness gradient, and treated with 30 µM ROCK inhibitor (Y-27632). Dotted lines in the upper panel represent channel boundaries. Scale bars, 10 µm in upper panel; 10 µm (horizontal) and 20 min (vertical) in lower panel.

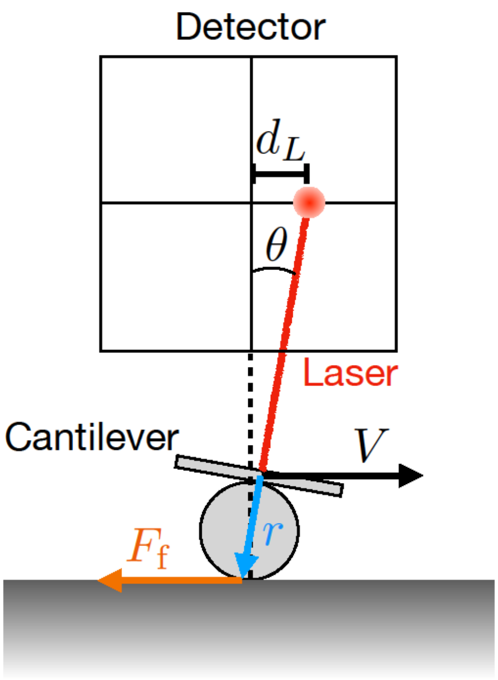

Supplementary Fig. 8. Illustration of friction measurements via LFM. The tipless AFM cantilever is functionalized with a 10 µm polystyrene bead and dragged back and forth along the surface at constant speed in a direction perpendicular to the cantilever axis. Friction between the bead and the surface creates a torsion on the cantilever that can be measured using the AFM's quadrant photodetector. If the cantilever slides at a velocity V over the surface, it experiences a friction force $F_f = \xi V$ on its contact point, where ξ is the friction coefficient. This force produces a torque on the cantilever: $\tau_f = F_f r = \xi V r$, where r is the lever arm. This frictional torque is balanced by the elastic torque arising from cantilever torsion: $\tau_e = k_\theta \theta \approx k_\theta d_L / h$, where k_θ is the cantilever's torsional stiffness, d_L is the movement of the laser on the photodetector, and θ is its torsion angle. The torsion angle relates to the lateral displacement d_L as $\sin\theta = d_L / h$, where h is the distance between the cantilever and the detector.

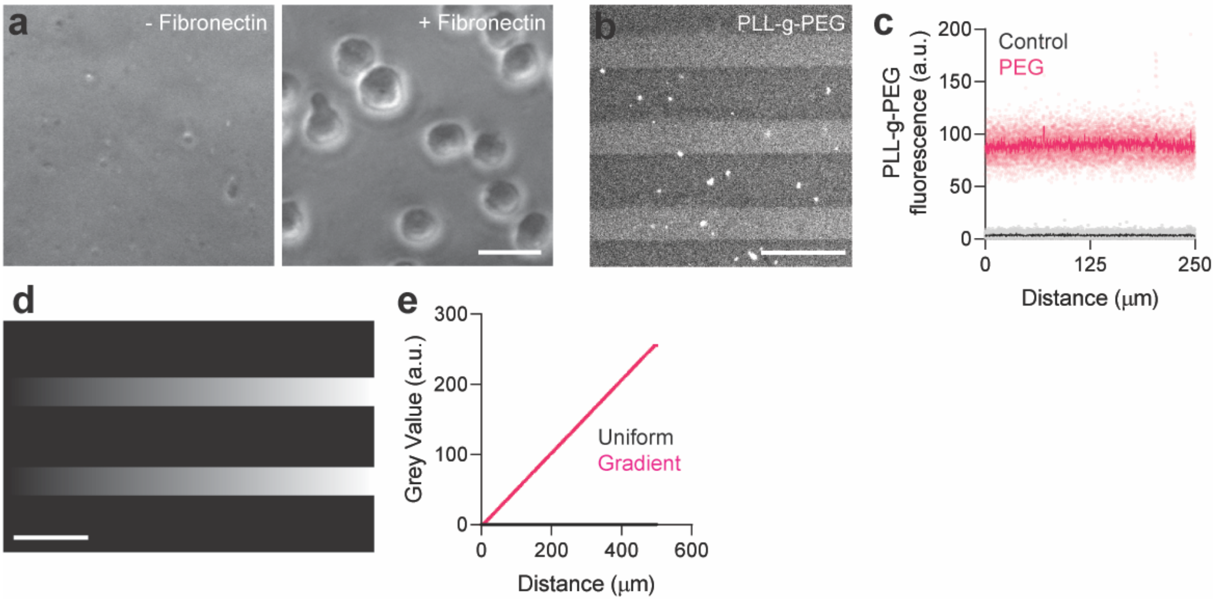

Supplementary Fig. 9. Agarose activation, PEG coating and photopatterning gradients for frictiotaxis experiments. a, Cells adhere to a fibronectin-coated agarose gel. Medium was aspirated and flushed several times to remove any cells in suspension. Scale bar, 20 µm. **b-c**, PLL-g-PEG/FITC-coated or uncoated (control) agarose microchannels (**b**) and quantification of fluorescence (**c**); n = 10; pale colours represent raw data; dark colours represent mean. Scale bar, 20 µm. **d**, The designed photopattern which exhibits alternating control and gradient masks. Scale bar, 100 µm. **e**, Quantification of (**d**)
