# Supplementary material for: Frictiotaxis underlies focal adhesion-independent durotaxis
Source: Nat Commun. 2025 Apr 23;16:3811. doi: 10.1038/s41467-025-58912-1 (PMC12019219; doi:10.1038/s41467-025-58912-1)
Supplement: Supplementary file 2 — Description of Additional Supplementary Information [file 41467_2025_58912_MOESM2_ESM.docx]

**Description of Additional Supplementary Files**

File Name: Supplementary Video 1

Description: Walker cell migrating within 10 µm width microchannels of 1% agarose, dispersed with fluorescent beads. Focal plane is at the top interface of the cell and the agarose channel wall. Movie length, 620 sec. Scale bar, 10 µm.

File Name: Supplementary Video 2

Description: Walker cells migrating within 10 µm width microchannels of 1% agarose. Movie length, 481 min. Scale bar, 100 µm.

File Name: Supplementary Video 3

Description: Walker cells undergo durotaxis without strong or specific adhesions to the substrate. Movie length, 40 min. Scale bar, 50 µm.

File Name: Supplementary Video 4

Description: Migration speed of Walker cells increases with higher substrate stiffness. Movie length, 110 min.

File Name: Supplementary Video 5

Description: Walker cells undergo durotaxis without integrin-based adhesions. Movie length, 40 min. Scale bar, 50 µm.

File Name: Supplementary Video 6

Description: In stiffness gradient and uniform stiffness microchannels, MyosinGFP accumulates at the cell rear and accumulation switches to the opposite pole when cells repolarise.

File Name: Supplementary Video 7

Description: Retrograde Myosin-GFP flow is observed in raw and registered timelapse sequences.

File Name: Supplementary Video 8

Description: Lifeact-GFP expressing Walker cells in microchannel reversing direction of migration and switching actomyosin flow. Intensity is shown as heatmap.

File Name: Supplementary Video 9

Description: Lifeact-GFP expressing Walker cells in microchannels. Upper panel: DMSO control. Lower panel: 30 µM Y-27632. Note that both cells in the lower panel do not form blebs and do not migrate.

File Name: Supplementary Video 10

Description: Numerical solution for the myosin concentration profile for a cell on uniform friction. The cell contracts symmetrically, breaks symmetry spontaneously and migrates to the right in this case.

File Name: Supplementary Video 11

Description: Numerical solution for the myosin concentration profile for a cell on uniform friction. The cell contracts symmetrically, breaks symmetry spontaneously and migrates to the left in this case.

File Name: Supplementary Video 12

Description: Numerical solution for the myosin concentration profile for a cell on a friction gradient, implemented as a linear friction profile. The cell contracts asymmetrically and it always migrates up the friction gradient (to the right).

File Name: Supplementary Video 13

Description: Walker cells undergo frictiotaxis on PEG-BSA gradients. Movie length, 98 min. Scale bar, 50 µm.
