# Supplementary material for: Frictiotaxis underlies focal adhesion-independent durotaxis
Source: Nat Commun. 2025 Apr 23;16:3811. doi: 10.1038/s41467-025-58912-1 (PMC12019219; doi:10.1038/s41467-025-58912-1)
Supplement: Supplementary file 16 — Reporting Summary [file 41467_2025_58912_MOESM16_ESM.pdf]

nature portfolio

Corresponding author(s): Roberto Mayor

Last updated by author(s): 2025/03/21

Reporting Summary

Nature Portfolio wishes to improve the reproducibility of the work that we publish. This form provides structure for consistency and transparency in reporting. For further information on Nature Portfolio policies, see our Editorial Policies and the Editorial Policy Checklist.

Statistics

For all statistical analyses, confirm that the following items are present in the figure legend, table legend, main text, or Methods section.

n/a	Confirmed	
☐	☒	The exact sample size (n) for each experimental group/condition, given as a discrete number and unit of measurement
☒	☐	A statement on whether measurements were taken from distinct samples or whether the same sample was measured repeatedly
☐	☒	The statistical test(s) used AND whether they are one- or two-sided<br>*Only common tests should be described solely by name; describe more complex techniques in the Methods section.*
☒	☐	A description of all covariates tested
☐	☒	A description of any assumptions or corrections, such as tests of normality and adjustment for multiple comparisons
☐	☒	A full description of the statistical parameters including central tendency (e.g. means) or other basic estimates (e.g. regression coefficient) AND variation (e.g. standard deviation) or associated estimates of uncertainty (e.g. confidence intervals)
☐	☒	For null hypothesis testing, the test statistic (e.g. F, t, r) with confidence intervals, effect sizes, degrees of freedom and P value noted<br>*Give P values as exact values whenever suitable.*
☒	☐	For Bayesian analysis, information on the choice of priors and Markov chain Monte Carlo settings
☒	☐	For hierarchical and complex designs, identification of the appropriate level for tests and full reporting of outcomes
☐	☒	Estimates of effect sizes (e.g. Cohen's d, Pearson's r), indicating how they were calculated

Our web collection on statistics for biologists contains articles on many of the points above.

Software and code

Policy information about availability of computer code

Data collection	Data was collected using FIJI and Microsoft Excel 360
Data analysis	Data was analysed using GraphPad Prism 9 and Origin 2023 for statistics and graph design. Cell tracks were analysed using the Chemotaxis and Migration Tool (Ibidi, Version 2.0). Images were generated using Adobe Illustrator.

For manuscripts utilizing custom algorithms or software that are central to the research but not yet described in published literature, software must be made available to editors and reviewers. We strongly encourage code deposition in a community repository (e.g. GitHub). See the Nature Portfolio guidelines for submitting code & software for further information.

Data

Policy information about availability of data

All manuscripts must include a data availability statement. This statement should provide the following information, where applicable:

- Accession codes, unique identifiers, or web links for publicly available datasets
- A description of any restrictions on data availability
- For clinical datasets or third party data, please ensure that the statement adheres to our policy

Source data are provided with this study. All other data are available in the main text or the supplementary materials.
The codes to solve the model equations and to fit it to the experimental data are available from the corresponding authors upon request and will be made publicly available upon acceptance of the manuscript.

Research involving human participants, their data, or biological material

Policy information about studies with human participants or human data. See also policy information about sex, gender (identity/presentation), and sexual orientation and race, ethnicity and racism.

Reporting on sex and gender	N/A
Reporting on race, ethnicity, or other socially relevant groupings	N/A
Population characteristics	N/A
Recruitment	N/A
Ethics oversight	N/A

Note that full information on the approval of the study protocol must also be provided in the manuscript.

Field-specific reporting

Please select the one below that is the best fit for your research. If you are not sure, read the appropriate sections before making your selection.

- [x] Life sciences
- [ ] Behavioural & social sciences
- [ ] Ecological, evolutionary & environmental sciences

For a reference copy of the document with all sections, see nature.com/documents/nr-reporting-summary-flat.pdf

Life sciences study design

All studies must disclose on these points even when the disclosure is negative.

Sample size	No statistical method was used to determine sample size, as sample size depended on availability of vital cells in the microchannel assay.
Data exclusions	Any excluded data are explained in the methods section. Selection criteria were cell survival and lack of interaction with other cells in the microchannels.
Replication	Each experiment was repeated at least three independent times (biological replicates) on different days.
Randomization	Parameters for each experiment were allocated to experimental groups and measured randomly.
Blinding	Authors were not blinded because of selection criteria (cell survival and lack of cell-cell interaction in microchannels), as stated in the methods section.

Reporting for specific materials, systems and methods

We require information from authors about some types of materials, experimental systems and methods used in many studies. Here, indicate whether each material, system or method listed is relevant to your study. If you are not sure if a list item applies to your research, read the appropriate section before selecting a response.

Materials & experimental systems

n/a	Involved in the study	
[ ]	[x]	Antibodies
[ ]	[x]	Eukaryotic cell lines
[x]	[ ]	Palaeontology and archaeology
[x]	[ ]	Animals and other organisms
[x]	[ ]	Clinical data
[x]	[ ]	Dual use research of concern
[x]	[ ]	Plants

Methods

n/a	Involved in the study	
[x]	[ ]	ChIP-seq
[x]	[ ]	Flow cytometry
[x]	[ ]	MRI-based neuroimaging

Antibodies

Antibodies used	Phospho-Paxillin (Tyr 118)_ThermoFisher_#44-722G (1:1000 dilution).<br>b1-integrin blocking antibody_clone AIIB2_Merck_#MABT409 (working concentration 10 µg/mL)<br>Goat-anti-rabbit IgG AlexaFluor 488_ThermoFisher_#A-11008 (1:500 dilution).

Validation	Phospho-Paxillin (Tyr 118) antibody was verified by cell treatment (according to the advanced verification procedure of the company), to ensure that the antibody binds to the antigen stated.<br>The b1-integrin blocking antibody was veryfied by cell treatment, with percentage of cell detachment upon treatment as readout.<br>The secondary antibody was commericially validated and has been widely in cell culture studies.

Eukaryotic cell lines

Policy information about cell lines and Sex and Gender in Research

Cell line source(s)	Walker-256-TC (RRID:CVCL_4984), Walker MLC-GFP, Walker Lifeact-GFP, and HL-60 cells (RRID:CVCL_0002) were a gift from Ewa Paluch (University of Cambridge, UK). Stable Walker MLC-GFP and Walker Lifeact-GFP cell lines were generated by transient transfection of WT Walker-256-TC cells followed by selection with 500 µg/ml G-418 and clonal separation by FACS, as described in Bergert et al., 2015 NCB.
Authentication	None was authenticated.
Mycoplasma contamination	Cell lines were tested negative for mycoplasma contamination using the MycoAlert Kit from Lonza.
Commonly misidentified lines (See ICLAC register)	No commonly misidentified cell lines per ICLAC register were used.

Plants

Seed stocks	N/A
Novel plant genotypes	N/A
Authentication	N/A
